# Supplementary material for: Clinical value of serum polyunsaturated fatty acids in patients with gastric polyps
Source: PeerJ. 2024 May 20;12:e17413. doi: 10.7717/peerj.17413 (PMC11114109; doi:10.7717/peerj.17413)
Supplement: Supplemental Information 1 [file peerj-12-17413-s001.doc]

**Operating procedures for the determination of polyunsaturated fatty acids**

**1 Purpose of Inspection**

This product uses tandem mass spectrometry to measure and evaluate the content of various fatty acids in human serum samples in vitro.

**2. Principles and Methods of Inspection**

Principle: First, protein precipitation liquid-liquid extraction is used to preprocess the serum sample, and then derivative reagents are added for fatty acid derivatization. The MRM mode of liquid chromatography tandem mass spectrometry is used for detection.

**3 Performance parameters**

**4 Sample Types**

This reagent kit is suitable for human serum samples, with a sample size requirement of 0.5-1.0 mL.

**5 Patient Preparation**

During sample collection, strict registration and accurate labeling should be carried out to ensure the accuracy of information for each sample. Collect blood from blood vessels without any additives or coagulants. Within 2 hours after blood collection, centrifuge at 1000-1200 rpm for 15 minutes and collect the supernatant.

**6 Types of containers and additives**

Coagulation promoting tube containing inert separation gel

**7 Instruments and reagents**

7.1 Instrument: Scienx API3200MD

7.2 Reagents:

The main components of this reagent kit are shown in Table 1:

Table 1 Main components of the kit

| **Reagent kit components** | **Noms** | **Numbers** |
| --- | --- | --- |
| calibrator-1 | 0.8 mL/bottle | 1 |
| calibrator-2 | 0.8 mL/bottle | 1 |
| calibrator-3 | 0.8 mL/bottle | 1 |
| calibrator-4 | 0.8 mL/bottle | 1 |
| calibrator-5 | 0.8 mL/bottle | 1 |
| calibrator-6 | 0.8 mL/bottle | 1 |
| Low value quality control products | 1.4 mL/bottle | 1 |
| High value quality control products | 1.4 mL/bottle | 1 |
| Internal standard products | 13 mL/bottle | 1 |
| Derivate-1 | 20 mL/bottle | 1 |
| Derivate-2 | 20 mL/bottle | 1 |
| Mobile phase additive A | 2 mL/bottle | 2 |
| Mobile phase additive B | 2 mL/bottle | 1 |

| Testing index | Concentration (umol/L) | | | | | | | |
| --- | --- | --- | --- | --- | --- | --- | --- | --- |
|  | S1 | S2 | S3 | S4 | S5 | S6 | QCL | QCH |
| C16:1 | 1.6 | 3.2 | 8 | 16 | 40 | 80 | 6 | 60 |
| C18:1 | 8 | 16 | 40 | 80 | 200 | 400 | 30 | 300 |
| C18:2 | 6 | 12 | 30 | 60 | 150 | 300 | 22.5 | 225 |
| α-C18:3 | 0.6 | 1.2 | 3 | 6 | 15 | 30 | 2.25 | 22.5 |
| γ-C18:3 | 0.2 | 0.4 | 1 | 2 | 5 | 10 | 0.75 | 7.5 |
| C20:1 | 0.2 | 0.4 | 1 | 2 | 5 | 10 | 0.75 | 7.5 |
| C20:4 | 1 | 2 | 5 | 10 | 25 | 50 | 3.75 | 37.5 |
| C20:5 | 0.2 | 0.4 | 1 | 2 | 5 | 10 | 0.75 | 7.5 |
| ω3-C22:5 | 0.2 | 0.4 | 1 | 2 | 5 | 10 | 0.75 | 7.5 |
| ω6-C22:5 | 0.2 | 0.4 | 1 | 2 | 5 | 10 | 0.75 | 7.5 |
| C22:6 | 0.4 | 0.8 | 2 | 4 | 10 | 20 | 1.5 | 15 |

**8 Environmental and Safety Control**

Environmental temperature: 15 ℃~30 ℃; Environmental humidity: not exceeding 80%;

Power supply: AC220V-240V, single-phase;

**9 Calibration Procedure**

9.1 Calibration material: kit kit matching reagents

9.2 Calibration method: Linear, full point calibration

9.3 Calibration frequency: Each batch

9.4 Traceability: NIST SRM1950

9.5 Storage conditions: The original packaging should be stored in a dark place at ≤ -18 ℃, with a validity period of 6 months.

After opening, store at -20 ℃ in dark for 15 days. Avoid repeated freeze-thaw cycles exceeding three times.

9.6 Preparation: Direct use.

**10 Quality Control**

10.1 Quality control substance: quality control substance matched with the reagent kit

10.2 Storage conditions: The original packaging should be stored in a dark place at ≤ -18 ℃, with a validity period of 6 months.

After opening, store at -20 ℃ in dark for 15 days.

Avoid repeated freeze-thaw cycles exceeding three times.

10.3 Quality control rules: Based on the bias (bias) in the national clinical laboratory quality evaluation report and the cumulative CV of indoor quality control, the following formula is used: σ= [(Tea | bias |)/CV], (where Tea is the allowed total error, bias and CV represent the bias and imprecision (coefficient of variation) observed by the testing program), calculate the bias and imprecision (coefficient of variation) for each test item σ Level, personalized design of quality control rules for testing items based on Westgard Sigma multiple rules.

10.4 Indoor Quality Control:

**11 Procedural steps**

1. Please read the user manual carefully before testing.

2. Before testing, restore the reagent kit and sample to room temperature.

3. Preparation of mobile phase before testing:

A-phase (aqueous phase) configuration:

Prepare mobile phase A or its equivalent in the ratio of mobile phase additive A: mobile phase additive B: deionized water: acetonitrile=1:1:950:50 (in ml);

B-phase (organic phase) configuration:

Prepare mobile phase B or its equivalent in the ratio of mobile phase additive A: mobile phase additive B: deionized water: acetonitrile=1:1:50:950 (in ml);

4. Preparation of test samples:

4.1. Sample preparation: Remove the calibrator, quality control sample, internal standard extraction solution, derivative, and test sample from -20 ℃, restore to room temperature, and vortex mix for 20 seconds before use;

4.2. Sample extraction: Take 100 calibrators, quality control samples, blank samples, and test samples each μ Add an internal standard solution of 100 to a 1.5mL centrifuge tube μ L. Add sample extraction liquid 600 μ L. Vortex mixing for 5 minutes, centrifuge at 13000 rpm for 5 minutes;

4.3. Sample transfer: Take 400% of the supernatant μ In a 96 well plate, blow dry with nitrogen at 40 ℃, and be careful not to blow the sample to an excessively dry state for a long time;

4.4. Sample Derivation-1: Add 150 μ L-derivative -1 solution, incubated at 500 rpm and 40 ℃ for 30 minutes;

4.5. Derivatives -2: Add 150 μ L-derivative -2 solution, incubated at 500 rpm and 40 ℃ for 15 minutes;

4.6. Sample detection: After standing for 8 hours, place the sample in a liquid chromatography tandem mass spectrometry injector for detection.

**12 Interference**

Hemolysis, lipolysis, jaundice, and EDTA-K2 can interfere with the results of sample determination.

**13 Result Calculation**

1) Calibration curve plotting: Using the concentration of the calibrator as the independent variable xi and the ratio of peak area of the corresponding concentration calibrator to the internal standard as the dependent variable yi, calculate the linear regression equation y=ax+b and the correlation coefficient r;

2) Quality control data analysis: When the calibration curve r ≥ 0.990, the signal strengths of low and high value quality control samples are inputted into the regression equation to obtain the concentration of quality control samples;

3) Serum sample data analysis: When the detection results of the quality control substance are within the expected range, the signal intensity of the serum sample is input into the regression equation to obtain the concentration of the target substance in the serum sample.
